# Infrared thermal mapping of osteotomy: influence of drill diameter and implant system

**DOI:** 10.3389/froh.2025.1660374

**Published:** 2025-10-03

**Authors:** Daniel Alvitez-Temoche, Mabel Huaman-De la Cruz, Berly Delgado-Cumpa, Gabriel Barriga-Yauri, Fran Espinoza-Carhuancho, Franco Mauricio, Ivan Calderon, Adrian V. Hernandez, Frank Mayta-Tovalino

**Affiliations:** 1 Vicerrectorado de Investigación, Faculty of Dentistry, Universidad Nacional Federico Villarreal, Lima, Peru; 2 Bibliometrics Evidence Evaluation and Systematic Reviews Group (BEERS) Human Medicine Career, Universidad Científica del Sur, Lima, Peru; 3 Academic Department, Universidad Nacional Mayor de San Marcos, Lima, Peru; 4 Unidad de Revisiones Sistemáticas y Meta-Análisis (URSIGET), Vicerrectorado de Investigación, Universidad San Ignacio de Loyola, Lima, Peru; 5 Health Outcomes, Policy and Evidence Synthesis (HOPES) Group, University of Connecticut School of Pharmacy, Storrs, CT, United States

**Keywords:** dental implant, *in vitro* study, surgical osteotomy, thermographic-infrared changes, bone

## Abstract

**Objective:**

Drill diameter and dental implant system influence heat generation during surgical osteotomy, affecting friction and temperature, which can impact osseointegration. The aim was to evaluate the influence of drill diameter, speed, and dental implant system on thermographic-infrared changes during surgical osteotomy.

**Materials and methods:**

An *in vitro* experimental design was used. The sample size was calculated using pilot study data, resulting in 120 specimens divided into three groups (*n* = 40) for each implant system (Arcsys, NeoBiotech, Osstem). The cow rib bones were prepared and disinfected with 10% sodium hypochlorite and 0.12% chlorhexidine, then dried with sterile gauze for 72 h. Implant systems were calibrated per manufacturer specifications. Surgical drilling was performed at 1,200 rpm and 40 Ncm torque using the Implantmed Plus motor. Thermographic measurements were taken using a Fluke TiS55 camera at specific bone points.

**Results:**

The Osstem system generated the highest temperature with the second drill, reaching 25.85 ± 1.97°C. The Arcys system showed a notable temperature increase from 23.52 ± 1.09°C to 25.57 ± 0.58°C with the second drill. NeoBiotech exhibited an increase in temperature from 22.75 ± 0.69 °C to 25.57 ± 1.72 °C following the use of the third drill. Statistical analysis indicated significant differences in both basal and pilot drill temperatures (°C) across three implant systems (*p* < 0.00 for both comparisons). Linear regression showed that basal temperature (*β* = −0.61°C, 95% CI: −0.88 to −0.34), pilot drill (*β* = 0.43°C, 95% CI: 0.17–0.69) and second drill (*β* = 0.76°C, 95% CI: 0.65–0.86) were significant predictors of final drilling temperature, while implant system had no significant effect. All temperature results are within safe limits for an osteotomy.

**Conclusion:**

The study found significant variations in temperature increase based on the implant system and drill diameter. Baseline temperature, pilot drill, and second drill temperatures were significant predictors of the third final drill temperature, while the implant system itself was not a significant factor.

## Introduction

Nowadays, modern dentistry seeks to provide esthetics, speech functionality, comfort and health to the patient in response to the various clinical conditions affecting the stomatognathic system. This may be achieved through interventions such as the removal of dental caries or the replacement of structurally compromised teeth (1).

Dental implants offer one of the best solutions to treat situations where teeth are missing, s and even circumstances where there are limitations to the use of conventional dental prostheses. In essence, the placement of an implant involves the creation of a hole in the bone and the subsequent insertion of the implant into the previously made preparation. The site where this procedure is performed is known as the osteotomy site (2).

Careful planning and precise placement of dental implants are crucial for maintaining the overall health of oral tissues (3). Recent advancements in cone beam computed tomography, dental scanning, and Computer-Aided Design/Manufacturing (CAD/CAM) technologies have significantly enhanced patient rehabilitation outcomes following clinical intervention (4). However, the main factor that determines the success of the treatment and the constant contact between the implant and the bone is the preservation of bone cell vitality. These cells are essential in the process of tissue healing and maturation (5).

Atraumatic surgical preparation of the implant site and a proper aseptic environment promote primary soft tissue and bone healing (1, 6). Importantly, temperature during surgical preparation is one of the variables that determines bone cell survival. In addition, friction between the cutting surface of surgical drills and bone is the predominant factor in temperature rise (1). Drilling depth, bone density and irrigation, as well as the design and material of the implant instruments, are other factors that affect temperature variations (7–9).

The use of bovine rib bone in experimental protocols is justified by its comparable biomechanical properties to human cortical bone, particularly in terms of density, elastic modulus, and structural response under drilling conditions. A variety of methods and tools exist to accurately gauge the heat produced during the preparation of implant sites. However, there are also tools that allow the temperature to be measured accurately and indirectly, using infrared thermography that detects changes in the surface through a color scale (10). Proper implant drilling will lead to an osseointegration process that will result in the formation of parallel fibers, bone tissue and lamellar bone. This will result in fully functional bone that will remodel throughout the patient's lifetime. Surpassing the critical thermal limit of 47°C for over 1 min can cause irreversible alterations in bone tissue, affecting vascular integrity and cellular viability. These changes interrupt the natural healing process and jeopardize successful osteointegration, potentially leading to implant failure (11).

There is limited evidence regarding the measurement of heat in the process of dental implant installation, as well as in the process of choosing the type of materials to be used. Therefore, this *in vitro* study aimed to assess the influence of drill diameter and dental implant system type on thermographic infrared variations observed during surgical osteotomy.

## Materials and methods

### Study design

The present investigation was an experimental *in vitro* study. Additionally, the Checklist for Reporting *in vitro* Studies (CRIS) guideline was used for writing the final manuscript for scientific publication (12). Thus, Clinical trial number is not applicable.

### Sample

**Figure 1:**
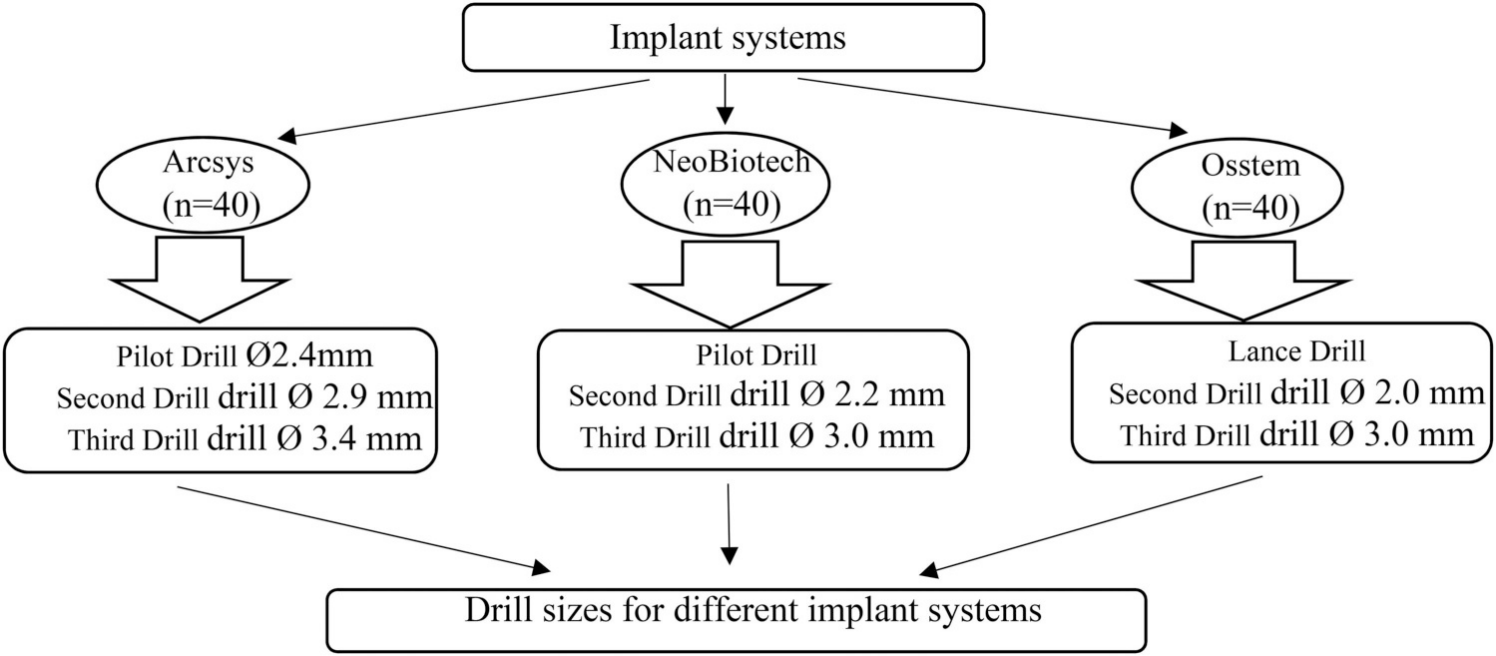
Groups formed according to diameter and implant system.

The sample size was determined based on pilot data using the mean comparison formula in Stata® 18, incorporating previously obtained means and standard deviations, with an alpha level of 0.05 and a statistical power of *β* = 0.8. Cow rib bones (*n* = 120) were randomly allocated into three equal groups (*n* = 40) corresponding to the implant systems: Arcsys, NeoBiotech, and Osstem. Simple random sampling was performed using a computer-generated sequence to ensure unbiased distribution across groups. Although randomization was applied during group allocation, the procedure was conducted without blinding of the operator due to the visible morphological differences among the implant systems (Figure 1).

### Inclusion criteria

The Arcsys, NeoBiotech and Osstem dental implant systems, selected for their wide acceptance and use in clinical practice, as well as for their diversity in design and technical characteristics, were included in this study. Dental implants with pilot drills, 2 mm drills and 3 mm diameter drills were considered to evaluate the impact of drill diameter on heat generation during osteotomy. Surgical osteotomy procedures included those involving dental implant placement, ensuring the clinical relevance of the results obtained. Cow rib bones of type D2 density were included, selected for their similarity to human bone density and their ability to provide relevant and applicable results to clinical practice.

### Exclusion criteria

Bone types other than cow bone were excluded to ensure consistency and comparability of results. In addition, thin ribs that did not provide sufficient bone material to perform the osteotomies effectively were excluded. Bones with fractures or deformities that could affect the accuracy of the measurements were also excluded. Bones showing signs of deterioration or decomposition were excluded to ensure the integrity of the bone material during the study. Finally, bones that could not be adequately monitored or measured with the available infrared thermography technology were excluded, ensuring the accuracy of the measurements.

### Bone procurement and characteristics

The cow rib bones were sourced from animals that were slaughtered for human consumption, which is unrelated to our investigation. These bones were obtained from the Frigorifico de Yerbateros slaughterhouse in Lima, Peru. Cow rib bone blocks specifically designed to resemble human bones in both trabecular and cortical structure were used. These blocks were standardized and selected to a type II bone density. The selection of these blocks was made due to their similarity to human bone density, which provides relevant and applicable results for clinical practice. In addition, they made sure to obtain the blocks from a specific batch to maintain consistency in the characteristics of the bone material used in the study.

### Bone preparation and antisepsis

The cow ribs were properly prepared and subjected to an antisepsis process with 10% sodium hypochlorite to remove any organic debris. In addition, 0.12% chlorhexidine was used to eliminate microorganisms. Subsequently, the samples were gently dried using sterile gauze and maintained at room temperature for 72 h to ensure complete evaporation and effective removal of potential contaminants based on prior protocols used in similar *in vitro* studies aiming to standardize surface conditions and minimize variability due to fluid content.

### Implant systems

Arcsys (Joinville, Brazil), NeoBiotech (Seoul, South Korea) and Osstem (Seoul, South Korea) dental implant systems were purchased and calibrated (Table 1). These systems were selected because of their wide acceptance and use in clinical practice, as well as their diversity in design and technical characteristics. Calibration of the implant systems was performed according to the manufacturer's specifications to ensure accuracy and consistency in the drilling procedures.

**Table 1:** Description of implant systems.

Brand	Company	City	Country	Technical features	Key differentiators
Arcsys	FGM Dental Group	Joinville	Brazil	- Customizable angle - Ti6Al4V titanium alloy (high mechanical strength) - Morse taper connection	- Innovative angle customization system for better adaptation - Suitable for complex procedures - Immediate load system
NeoBiotech	NeoBiotech Co., Ltd.	Seoul	South Korea	- Internal submerged design - S.L.A. treated surface (sandblasted and acid-etched) - Internal hexagonal connection - High primary stability	- Surface designed for rapid osseointegration, ideal for patients with low-density bone
Osstem	Osstem Implant Co., Ltd.	Seoul	South Korea	- SA surface (alumina sandblasted and acid-etched) - Tapered body design for high initial stability - Variety of diameters and lengths	- Wide range of options for different surgical needs - Globally recognized for reliability in dental rehabilitation

### Bone drilling protocol for implants

**Figure 2:**
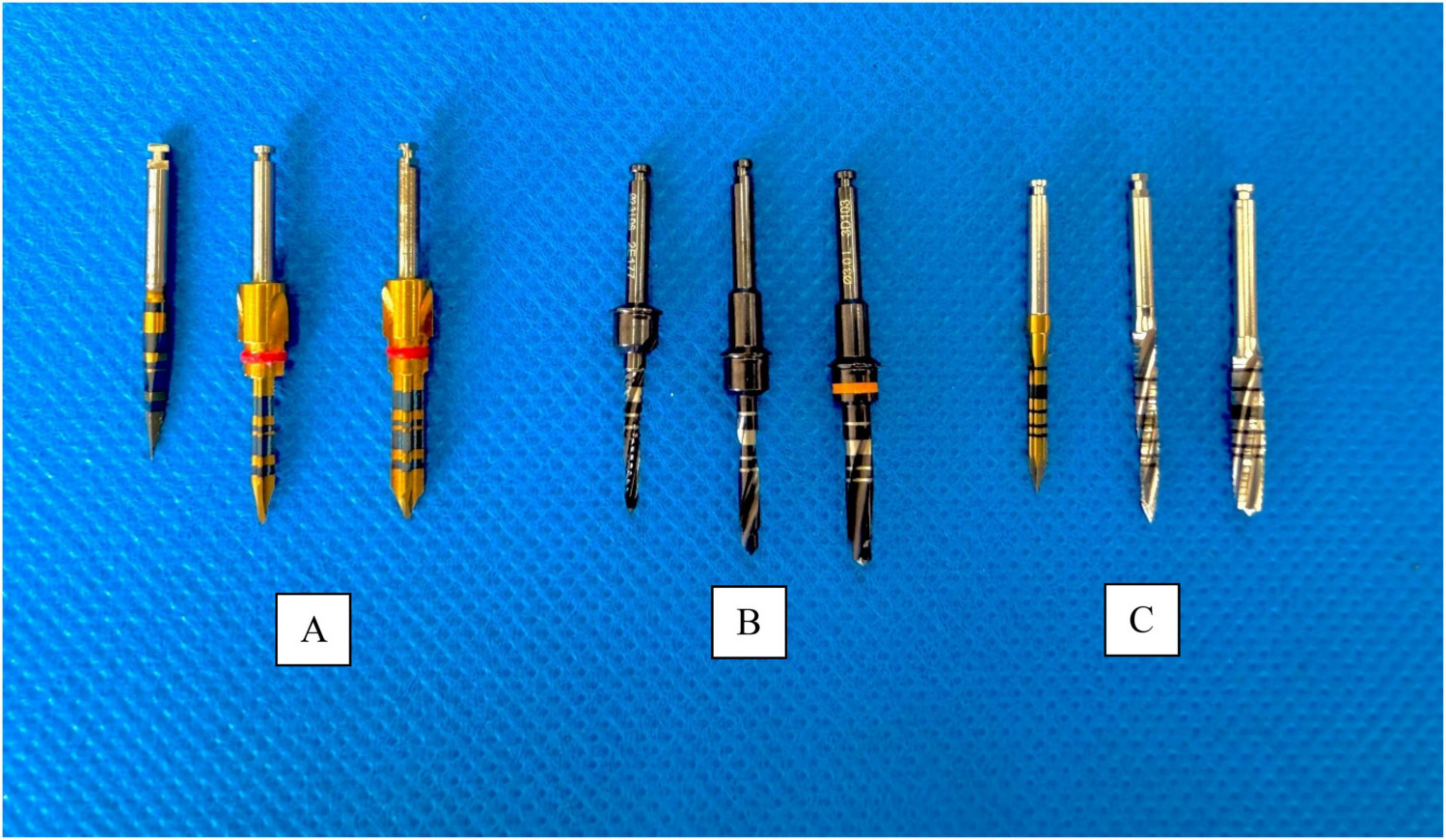
Drilling sequence of the different implant systems. **(A)** Arcsys drills. **(B)** NeoBiotech drills. **(C)** Osstem drills.

Surgical preparation was performed at 1,200 rpm and 40 Ncm torque using the Implantmed Plus implant motor (W&H, Bürmoos, Austria). A sequential bone-surgical drilling protocol was followed, using a Lance Drill, second drill and a third drill for all three dental implant systems. For all samples, it was decided not to use irrigation so as not to alter the heat generation during the surgical drilling protocol. The drilling depth was standardized at 10 mm for all osteotomies. Debris were carefully removed with sterile gauze between each drilling step to avoid build-up and possible interference with subsequent osteotomies. Although the set up did not replicate clinical irrigation, the research allowed for controlled assessment of thermal thresholds illustrating the importance of cooling strategy while controlling for all other variables (Figure 2).

### Thermographic measurement with the Fluke camera

During the entire drilling process, the temperature was measured using a Fluke TiS55 thermographic camera (Everett, Washington, USA). This high-resolution camera allowed accurate measurements of temperature changes in degrees Celsius (°C) during the surgical osteotomy. Measurements were taken at three specific points on the bone: on the side opposite the osteotomy, 1 mm below the drilling site and 5 mm below the drilling site. All the captures made by the thermographic camera were taken in photo format, collecting the drilling time until the projected depth was reached. Using the video editing tool of the SmartView V 4.0 software, each image was analyzed using a frame-by-frame analysis, collecting the drilling time and temperature until the projected depth was reached. All procedures were conducted under controlled ambient conditions at a temperature of 18°C. Thermographic images were acquired using a factory-calibrated Fluke thermal imaging system, with calibration performed by a certified specialist engineer to ensure measurement accuracy and reliability throughout the experimental procedures.

### Analysis plan

Stata® 18 statistical software was used. Measures of central tendency and dispersion such as mean, median, standard deviation and interquartile range were calculated for numerical variables such as diameter and thermographic-infrared changes. Then, the Shapiro–Wilk test and box-plot plots were applied to evaluate normality and the homogeneity of variances test. In addition, the Kruskall-Wallis test was used for inferential statistics. Finally, a linear regression analysis was performed to predict the value of the dependent variable (thermographic-infrared change) based on the value of the other independent variables (or predictor variables).

## Results

**Figure 3:**
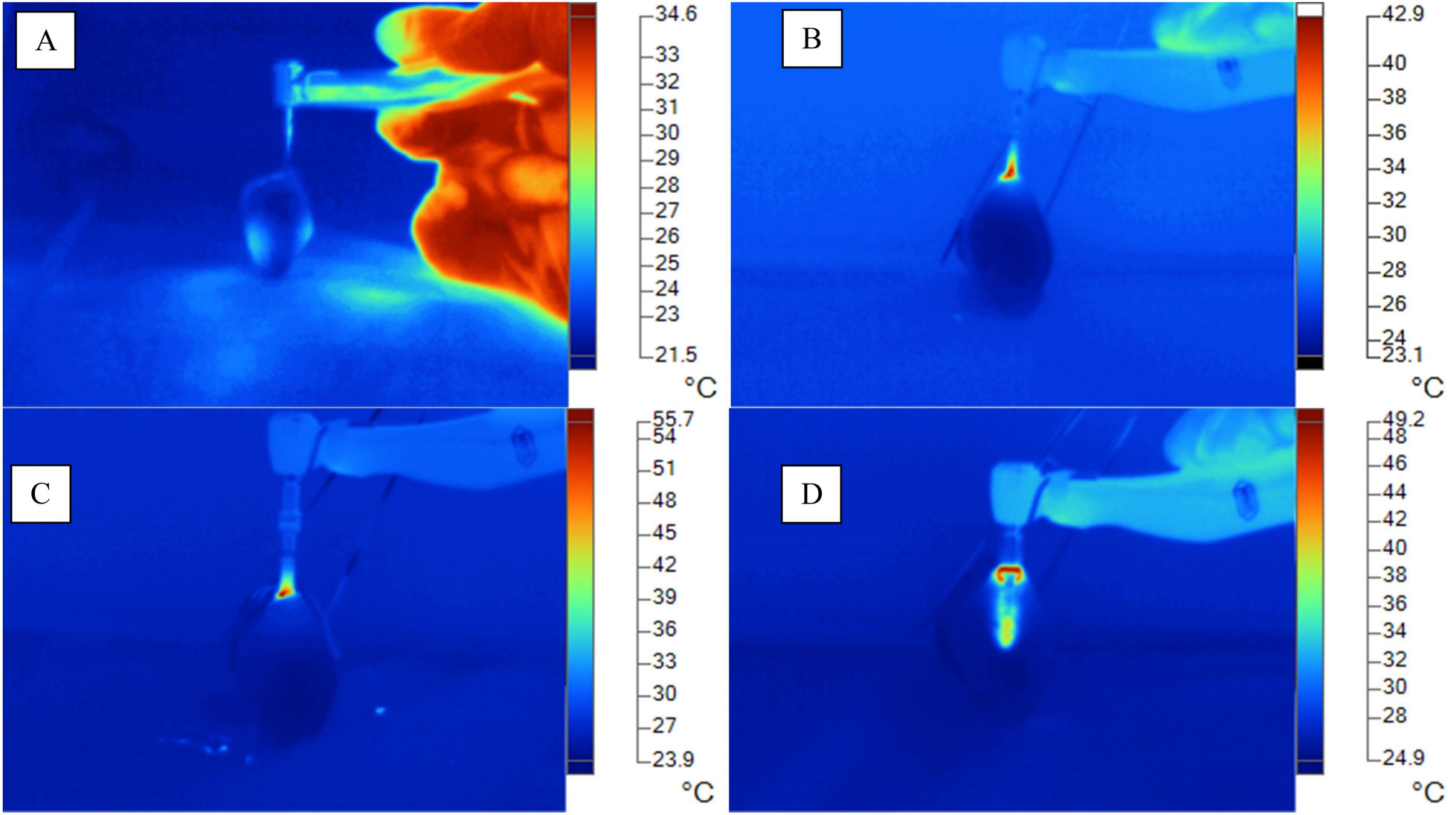
Thermographic-infrared changes during surgical osteotomy (°C). **(A)** Basal temperature (°C). **(B)** Arcsys Temperature (°C). **(C)** NeoBiotech Temperature (°C). **(D)** Osstem temperature (°C).

It was observed that the Arcys system presented a basal temperature of 23.52 ± 1.09°C, which increased to 25.13 ± 0.69°C with the pilot drill, to 25.57 ± 0.58°C with the 2 mm drill and to 25.43 ± 1.01°C with the 3 mm drill. On the other hand, the NeoBiotech system showed a basal temperature of 22.75 ± 0.69°C, which increased to 23.8 ± 0.85°C with the pilot drill, to 24.48 ± 1.71°C with the 2-mm drill and to 25.57 ± 1.72°C with the 3-mm drill. Finally, the Osstem system had a basal temperature of 23.05 ± 0.94°C, which increased to 23.45 ± 1.34°C with the pilot drill, to 25.85 ± 1.97°C with the 2-mm drill and to 24.97 ± 1.63°C with the 3-mm drill. These results indicate variations in temperature rise depending on the implant system and the diameter of the drill used. Statistically significant differences (*p* < 0.05) were observed exclusively in baseline temperature (°C) and pilot drill temperature (°C) across the three evaluated dental implant systems (Figure 3; Table 2).

**Table 2:** Comparison of diameter, speed and dental implant system on thermographic-infrared changes during surgical osteotomy.

System	Baseline °C		Pilot drill °C		Second drill °C		Third drill °C		*p* *
	Mean ± SD		Mean ± SD		Mean ± SD		Mean ± SD		
Arcsys^a^	23.52	1.09	25.13	0.69	25.57	0.58	25.43	1.01	<0.00
NeoBiotech^b^	22.75	0.69	23.8	0.85	24.48	1.71	25.57	1.72	<0.00
Osstem^c^	23.05	0.94	23.45	1.34	25.85	1.97	24.97	1.63	<0.00
*p* **	0.00		0.00		0.05		0.06		

a Arcsys: Titanium nitride coated drills, the drilling sequence was pilot drill Ø2.4 mm, the next drill Ø2.9, and Ø3.4 mm.

b NeoBiotech: The initial drill is called Lindermann Ø2.0, Ø2.2 and Ø3.0 mm.

c Osstem: The initial drill is called LanceDrill, Ø2.2 and Ø3.0 mm.

* *p* Shapiro Wilk Normality Test.

** *p* Kruskall-Wallis test.

**Figure 4:**
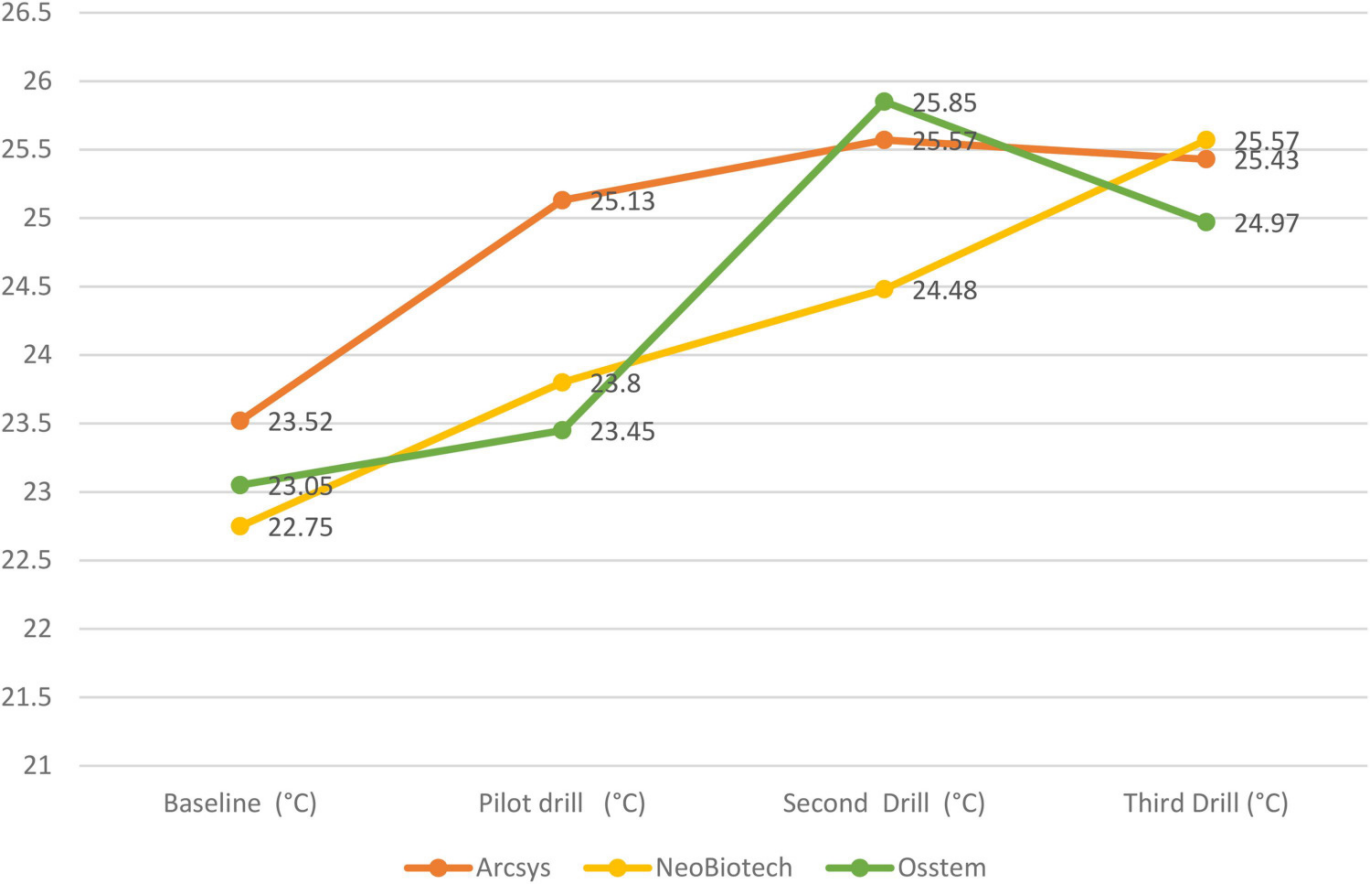
Influence of dental implant diameter and system on thermographic-infrared changes.

The graph shows the following trends in the temperatures recorded during surgical drilling for the different dental implant systems. The Arcsys system shows an increase in temperature from a baseline of 23.52°C–25.13°C with the pilot drill, 25.57°C with the 2 mm drill and 25.43°C with the 3 mm drill. On the other hand, the NeoBiotech system shows an increase in temperature from 22.75°C at baseline to 23.8°C with the pilot drill, 24.48°C with the 2 mm drill and 25.57°C with the 3 mm drill. Finally, the Osstem system records an increase in temperature from 23.05°C at baseline to 23.45°C with the pilot drill, 25.85°C with the 2 mm drill and 24.97°C with the 3 mm drill. These trends reflect how the temperature varies as a function of the implant system and the diameter of the drill used. Of the three systems, the Osstem system generates the highest temperature when drilling the bone, reaching 25.85°C with the 2 mm drill (Figure 4).

For every degree that Baseline temperature (Baseline °C) increased, the final drill temperature (Third Drill °C) decreased by 0.61°C (95% CI: −0.88 to −0.34) and this was significant (*p* < 0.00). For every degree the Pilot drill °C increases, the final drill temperature (Third Drill °C) increases by 0.43°C (95% CI: 0.17–0.69) and this was significant (*p* = 0.00). For every degree that Second drill (Second Drill °C) temperature increases, the final drilling temperature (Third Drill °C) increases by 0.76°C (95% CI: 0.65–0.86) and this was significant (*p* < 0.00). There were no significant differences for the implant system (Implantmed) in final drill temperature (Third Drill °C) (*p* = 0.39). These results show that the basal, pilot and second drill temperatures are relevant in predicting the final third mill temperature, while the implant system made no significant difference (Table 3).

**Table 3:** Linear regression of final drilling (third drill °C) according to baseline °C, pilot drill °C, second drill °C and implant system.

Variables	Third drill °C
	*β*
Baseline °C	−0.61°C (IC 95%: −0.88 to −0.34)
Pilot drill °C	0.43°C (IC 95%: 0.17 to 0.69)
Second drill °C	0.76°C (IC 95%: 0.65 to 0.86)
Implant system	−0.11°C (IC 95%: −0.36 to 0.14)

Arcsys: Titanium nitride coated drills, the drilling sequence was pilot drill Ø2.4 mm, the next drill Ø2.9, and Ø3.4 mm.

NeoBiotech: The initial drill is called Lindermann Ø2.0, Ø2.2 and Ø3.0 mm.

Osstem: The initial drill is called LanceDrill, Ø2.2 and Ø3.0 mm.

## Discussion

The success of implant surgery depends largely on reducing thermal damage during osteotomy preparation (13). Excessive temperature generated during implant drilling could cause tissue necrosis, compromising the healing of the surrounding bone (14). Factors influencing heat generation during osteotomy include drill diameter, drill design and material, irrigation type, speed, drilling time and bone density, etc. (15). Therefore, this *in vitro* study investigated how drill diameter, speed and dental implant system influence thermographic and infrared changes during surgical osteotomy.

In addition, it was decided in this investigation not to use saline irrigation during osteotomy to avoid altering heat generation and thus to be able to accurately measure thermal changes with the thermographic camera. The absence of irrigation allowed us to obtain more accurate data on the temperature generated during drilling, which is crucial to evaluate the impact of different variables, such as drill diameter and drilling speed, on heat generation. In this study, surgical preparation was performed at 1,200 rpm using the Implantmed Plus implant motor (W&H, Bürmoos, Austria). In addition, a sequential bone-surgical drilling protocol was followed, using a Lance Drill, a 2 mm Drill and a 3 mm Drill for all three dental implant systems.

The present *in vitro* study showed a progressive increase in temperature with drill diameter, especially in the Arcys and Osstem systems, where the temperature increased from 25.13°C (pilot drill) to 25.57°C (2 mm drill) and then decreased slightly with the 3 mm drill. The linear regression analysis revealed clinically relevant associations that inform best practices in implant surgery. Specifically, lower baseline bone temperatures were linked to reduced thermal output during final drilling, suggesting that initiating osteotomy under cooler conditions may help prevent excessive heat buildup. Conversely, increases in temperature during the pilot and second drilling stages were positively correlated with higher final drill temperatures, underscoring the cumulative thermal effect of early-stage instrumentation. These findings highlight the importance of careful thermal management throughout each drilling phase to safeguard bone integrity and promote optimal conditions for successful osseointegration.

Similar findings were found in the study by Limmeechokchai et al. which investigated the heat and sound generated during implant osteotomy using different types of drills in artificial and bovine bone blocks. Osteotomies were performed with four drilling systems: N1 (OsseoShaper), NobelActive, V3 (MIS), and BLX (Straumann), measuring changes in temperature, drilling time, and sound generated. The results showed that OsseoShaper produced lower temperatures and less sound compared to other systems, although it required more drilling time. It was concluded that OsseoShaper is effective in reducing heat and sound during osteotomy, even without irrigation. Also, our results indicated variations in temperature rise depending on the implant system and the diameter of the drill used. Of the three systems, the Osstem system generated the highest temperature when drilling the bone, reaching 25.85°C with the 2 mm drill (16).

Furthermore, Marzook et al. in their investigations on the impact of implant drilling speed on heat generation and bone viability at osteotomy sites in rabbits observed that groups with higher speeds showed less heat generation compared to lower speed groups. Therefore, they concluded that drilling at higher speeds in dense bone and with irrigation produces less heat (17). Similarly, in the study conducted by Salomó-Coll et al., the impact of bone type, drill diameter, drilling speed, and irrigation on heat generation during osteotomies for dental implants was assessed. They used polyurethane foam blocks to simulate type I (dense) and type IV (soft) bone, with drills of 2 and 3.5 mm diameters, at various speeds (50, 100, and 800 rpm), both with and without irrigation. The findings indicated that the most significant temperature difference occurred at 100 rpm without irrigation and at 800 rpm with irrigation. Importantly, the maximum temperatures remained below the critical threshold for causing osteonecrosis. Likewise, our results showed the variations in temperature according to the diameter of the drills and the implant system (18).

In the research by Frösch et al., they compared the heat generation during the preparation of guided osteotomies (GOP) with a conventional approach (CA) using single and sequential drilling protocols. The authors reported that GOP generated significantly higher temperatures than CA in the diameters of 2.2, 3.5 and 4.2 mm. Therefore, they concluded that sequential drilling resulted in greater heat generation and longer duration of latent heat than single drilling. In the case of our research, a sequential procedure was used, using a Lance Drill, a 2 mm Drill and a 3 mm Drill for the three dental implant systems, which probably influenced the results and resulted in greater heat generation (5).

In the *in vitro* study by Guler et al., they evaluated the thermal variations during osteotomy with three different implant systems (Implantium®, Straumann® and Anyridge®) at different drilling speeds in bovine bone. In addition, the maximum heat generated in each system was measured at speeds of 150, 250 and 400 rpm, where the results obtained showed a significant increase in temperature at 250 and 400 rpm in most of the drills, with the maximum heat recorded being 59.37°C for Implantium, 58.71°C for Straumann and 75.67°C for Anyridge. Therefore, they concluded that the temperatures were within clinically safe limits. In contrast, with the present study that only used 1,200 rpm in the three implant systems Arcsys (Joinville, Brazil), NeoBiotech (Seoul, South Korea) and Osstem (Seoul, South Korea). Therefore, the temperatures reached were considerably lower, with maximum values of 25.85°C for the Osstem system, 25.57°C for NeoBiotech and 25.57°C for Arcsys. This suggests that the speed range used in the study by Guler et al. might have influenced heat generation more compared to the single speed range of 1,200 rpm in this study (19).

On the other hand, the study by Koutiech et al. focused on the differences between drilling techniques and the importance of irrigation to control the heat generated. Drilling was performed at 1,500 rpm and with constant irrigation. The authors observed that gradual drilling generated a significantly higher temperature increase than single drilling. In contrast, our research focused more on the influence of drill diameter and basal temperature. The key difference was found in the use of irrigation in the study, which probably contributed to reducing the heat generated, while in our study it showed the effects of lack of irrigation, which could explain the differences in the resulting temperatures (20). Another result that contrasted with our findings was the *in vitro* study by Marzook et al., who showed that increasing the drill diameter also reduces heat generation compared to smaller diameter drills (17).

One of the main strengths of our *in vitro* study was that it evaluated variables such as implant type, speed, and drill diameter, ensuring results applicable to clinical practice. Also, the use of the Fluke thermal imaging camera provided us with accurate and detailed measurements of thermal variations during drilling, which allowed an analysis of the impact of drill diameter on heat generation. In addition, three different implant systems were used for greater representativeness, and finally, tests such as linear regression were included in the statistical analysis. However, the study presented some limitations such as the use of an *in vitro* model, which may not accurately reflect the real conditions of an *in vivo* procedure. In addition, there may be variations in the results when using bovine bone instead of human bone, and important factors such as irrigation and drilling pressure, which affect the temperature generated during osteotomy, may have been omitted. Other limitations include the small sample size, environmental conditions, and operator experience. While bovine bone serves as a valuable surrogate in experimental settings, the absence of soft tissue simulation may influence how heat dissipates during drilling.

The current study used various methodologies to standardize experimental conditions and clarify important variables that pertain to thermal generation, specifically temperature, during osteotomy. Therefore, we used a 72-h bone dehydration period as opposed to hydration in NBF (neutral buffered formalin), like previous *in vitro* research. However, we recognize that dehydrating the bone increased its water content, which we consider a limitation. Also, the drilling speed was fixed at 1,200 rpm for all instruments, as our intent was to standardize the variable related to bone density and mitigate the negative consequences that temperature effects could have, using constant pressure applied by a single operator that was trained in oral implantology. Irrigation was also excluded in order to develop extreme thermal situations and to contrast the effect of heat generated by friction from routine waste between each drilling. Finally, the depth of the drilling was standardized to 10 mm (approximately average for implant length), allowing for comparison between samples.

This limitation reminds us that *in vivo* conditions are more complex, and future research should strive for models that better reflect clinical realities. Finally, the current findings indicate no statistically significant influence of the implant system on final drilling temperature, this should not discourage further investigation. The limited availability of comparative studies makes it difficult to rule out potential effects entirely. Future research could explore how variations in implant macrodesign, surface characteristics, and material composition might impact heat generation and dissipation, especially in diverse surgical scenarios. Deepening our understanding in this area could help refine thermal safety strategies and support clinicians in making more informed, patient-centered implant choices.

## Conclusion

The study showed that variations in osteotomy temperature rise depended on the implant system and the diameter of the drill used. Although the Arcsys, NeoBiotech and Osstem systems had different temperature rise patterns, the Osstem system showed the highest recorded values under the tested conditions. The basal temperature and the pilot and 2 mm drills were found to be significant predictors of the final temperature, unlike the implant system itself, which did not have a significant result. The authors emphasize the importance of controlling drilling variables to avoid thermal temperatures in bone tissue in dental implant surgery. Further *in vivo* validation is needed (21), as the thermal behavior observed in clinical settings involves dynamic interactions that *in vitro* models can only partially mimic. Confirming these patterns under real physiological conditions would help ensure safer surgical protocols and support more informed, patient-centered decision making.

## Data Availability

The raw data supporting the conclusions of this article will be made available by the authors, without undue reservation.
